# Nontypeable *Haemophilus influenzae* exploits the interaction between protein-E and vitronectin for the adherence and invasion to bronchial epithelial cells

**DOI:** 10.1186/s12866-015-0600-8

**Published:** 2015-11-14

**Authors:** Masaki Ikeda, Noriyuki Enomoto, Dai Hashimoto, Tomoyuki Fujisawa, Naoki Inui, Yutaro Nakamura, Takafumi Suda, Toshi Nagata

**Affiliations:** Second Division, Department of Internal Medicine, Hamamatsu University School of Medicine, Hamamatsu, Japan; Department of Clinical Pharmacology and Therapeutics, Hamamatsu University School of Medicine, Hamamatsu, Japan; Department of Health Science, Hamamatsu University School of Medicine, Hamamatsu, Japan

**Keywords:** *Haemophilus influenzae*, NTHi, Intracellular invasion, Protein-E, Vitronectin

## Abstract

**Background:**

Nontypeable *Haemophilus influenzae* (NTHi) is one of the most common Gram-negative pathogens in otitis media and exacerbation of chronic obstructive pulmonary disease. NTHi has been reported to invade bronchial epithelial cells. This penetration enables NTHi to evade the host immune system and antibiotics, and it seems to be related to the intractable features of these diseases. However, the precise mechanism of the invasion has been unknown. We hypothesized that protein-E, an outer membrane protein of NTHi, plays a role in this penetration into bronchial epithelial cells.

**Results:**

We utilized two NTHi strains. NTHi efficiently attached to plate-bound vitronectin (254–309 / field at 1,000× magnification) and this attachment was blocked by pretreatment with protein-E peptide (PE^84–108^). The blockade of adhesion was dependent on the concentration of PE^84–108^. NTHi strains invaded bronchial epithelial cells and the intracellular bacteria were localized in early endosomes. Furthermore, intracellular invasion of NTHi was also blocked by PE^84–108^, but not by Arg-Gly-Asp (RGD) peptide. Pretreatment with PE^84–108^ significantly prevented cells from being invaded by both NTHi strains, which was confirmed by fluorescent microscope observation. In addition, pretreatment with PE^84–108^ significantly reduced percentages of CFU after gentamicin treatment of cells per input CFU.

**Conclusions:**

These results suggest that NTHi does not directly bind to the cell surface, but binds to host vitronectin that is bound to the cell surface, via bacterial protein-E. Bacterial protein-E and host vitronectin play a role in the attachment to bronchial epithelial cells and is also involved in the subsequent intracellular invasion of NTHi. A novel vaccine or treatment strategy targeting the protein-E-vitronectin axis may prevent respiratory intracellular infection of NTHi and may lead to better clinical outcomes.

**Electronic supplementary material:**

The online version of this article (doi:10.1186/s12866-015-0600-8) contains supplementary material, which is available to authorized users.

## Background

*Haemophilus influenzae (H. influenzae)* is a Gram-negative bacterium and is one of the most prevalent pathogens worldwide. Some *H. influenzae* strains have a polysaccharide capsule and they are divided into six serotypes (a-f), termed typeable *H. influenzae*. The other strains do not possess a capsule, and they are termed nontypeable *H.influenzae* (NTHi). NTHi is a major pathogen of mucosal infections such as otitis media and exacerbation of chronic obstructive pulmonary disease (COPD) [1, 2]. Substantial numbers of COPD patients are colonized by NTHi in their lower airways, and　this type of bacteria frequently causes chronic bronchitis and acute exacerbation of COPD [3].

NTHi can invade host bronchial epithelial cells, and this invasion enables NTHi to escape from host immune system [4, 5]. Intracellular NTHi is able to evade high concentration of antibiotics and becomes clinically intractable [6, 7]. Therefore, preventing NTHi from invading epithelial cells is crucially important for the prophylaxis and treatment of diseases mentioned above. However, the exact mechanism by which NTHi breaks into bronchial epithelial cells has been unknown.

To penetrate into bronchial epithelial cells, adherence of NTHi to these cells is essential. Previous studies reported the significance of adhesion molecules for the direct attachment of NTHi to epithelial cells [8, 9–11]. Some of these adhesion molecules on NTHi such as high-molecular-weight proteins (HMW1 and 2) possess Arg-Gly-Asp (RGD) sequence [12], and this RGD sequence can bind to integrin-receptors on epithelial cell surface [11].

In addition, vitronectin, which is in plasma and extracellular matrix, also binds to NTHi and is related with its adhesion to cells [13]. A recent report showed that protein-E (gene name *pe*, HI 0178 in Rd KW20 strain, NTHI 0267 in 86-028NP strain), a NTHi outer membrane protein binds vitronectin and is related to NTHi serum resistance [14]. Vitronectin possesses three heparin-binding domains (HBDs) [15] and the C-terminal HBD-3 corresponds to a protein E binding region [16]. Vitronectin also has RGD sequence which binds to integrin receptors on epithelial cell surface [15]. However, the role of protein-E and vitronectin in the intracellular invasion of NTHi has not been fully elucidated.

In the present study, we demonstrated that intracellular invasion of NTHi into bronchial epithelial cells is dependent on protein-E via its binding with vitronectin. To our knowledge, this is the first report to show that protein-E not only plays a role in the attachment to epithelial cells but also is involved in the subsequent intracellular invasion of NTHi. The protein-E-vitronectin axis may become a novel therapeutic and vaccine target for NTHi infection.

## Methods

### Bacterial strains and cell culture

Two strains of NTHi were used in this study. One was NTHi clinical isolate HUSM 0481, which was cultured from the sputum of a patient with community-acquired pneumonia at Hamamatsu University Hospital in Hamamatsu, Japan. The sample was taken as part of standard care. The other was a commercially available NTHi strain ATCC 19418 (American Type Culture Collection (ATCC), Manassas, VA). NTHi was precultured in brain heart infusion (BHI) liquid broth supplemented with NAD and hemin (both at 1 μg/ml) and cultured overnight on chocolate agar plates at 37 °C.

*Escherichia coli* (*E. coli*, strain Le392) and *Listeria monocytogenes* (*L. monocytogenes*, strain 10403 s) were precultured in BHI.

BEAS-2B cells (ATCC), a human bronchial epithelial cell line, were cultured on glass-bottomed dishes in LHC-8 medium without gentamicin (Life technologies/Gibco, Carlsbad, CA) containing 500 ng/ml of epinephrine (Sigma-Aldrich, St. Louis, MO) and 0.1 ng/ml of retinoic acid (Sigma-Aldrich).

### Infection with bacteria and evaluation of their penetration into BEAS-2B cells

Confluent BEAS-2B cells on glass-bottomed dishes were infected with several types of bacteria at a multiplicity of infection (MOI) of 100 for 2 hours at 37 °C with 5 % CO_2_. After killing any extracellular bacteria with a 2-hour treatment of 100 μg/ml gentamicin (Sigma-Aldrich) and washing 3 times, epithelial cells and bacteria were stained with the mixture of 1.5 μl of 3.34 mM SYTO 9 and 1.5 μl of 20 mM propidium iodide per 2 ml of medium (LIVE/DEAD® BacLight bacterial viability kit, Invitrogen/Molecular Probes, Eugene, OR) for 15 minutes according to the manufacturer’s instructions, and then stained with 10 μg/ml of Hoechst 33342 (Hoechst, Invitrogen/Molecular Probes) for 30 minutes to evaluate the invaded cells. The numbers of cells with one or more intracellular bacteria were counted with a fluorescent microscope (BZ-9000; Keyence, Osaka, Japan). One hundred cells were counted three times at different sites, at a magnification of 1,000×, and the percentage of invaded cells was calculated. For the evaluation of viable intracellular bacteria, cells were lysed with distilled water, after killing of extracellular bacteria with gentamicin and washing 3 times as described above, and the bacteria were cultured on chocolate-agar plates overnight at 37 °C. Then, the percentage of colony number after gentamicin treatment per input bacterial number was calculated.

### Immunofluorescent staining and evaluation of NTHi localization in BEAS-2B cells

After infection with NTHi and treatment with gentamicin to kill the extracellular bacteria, cells were fixed with 4 % paraformaldehyde phosphate (4 % PFA, Wako, Osaka, Japan) for 15 minutes at room temperature. Specimens were incubated with 1 % BSA in PBS for 30 minutes and washed with PBS three times. Early endosomes were stained with goat anti-human EEA1 (N-19) antibody (Santa Cruz Biotechnology, Dallas, TX). Late endosomes were stained with mouse monoclonal anti-human LAMP-1 (H4A3) antibody (Santa Cruz Biotechnology). As for the staining of acidic endosomes, after staining of viable bacteria with LIVE/DEAD® without 4 % PFA, acidic endosomes were stained with LysoTracker® Red (Molecular Probes/Life Technologies, Carlsbad, CA). Nuclei were stained with Hoechst. After staining, micrographs were taken with a fluorescent microscope (BZ-9000).

### Adhesion of NTHi to immobilized vitronectin

Vitronectin from human plasma (0.1 μg/cm^2^; Sigma-Aldrich) was incubated on glass-bottomed dishes at 37 °C for 2 hours. Bovine serum albumin (BSA; 0.1 μg/cm^2^; Sigma-Aldrich), as a negative control, was also incubated on glass-bottomed dishes. In some experiments, 1,000 μg/ml of heparin (Sigma-Aldrich) or 100 μg/ml of protein-E peptide (PE^84–108^; MBL, Nagoya, Japan) was incubated with plate-bound vitronectin for 60 minutes before NTHi incubation. PE^84–108^ peptide was synthesized based on the predicted amino acid sequence from HI 0178 [17]. NTHi was incubated on the dishes for 30 minutes, and the dishes were washed with PBS three times. Attached NTHi were stained with LIVE/DEAD® for 15 minutes, and the number of bacteria was counted with a fluorescent microscope (BZ-9000) at a magnification of 1,000 × .

### Detection of vitronectin on BEAS-2B Cells

Confluent BEAS-2B cells on glass-bottomed dishes were fixed with 4 % paraformaldehyde phosphate for 15 minutes at room temperature. Cells were incubated with 1 % BSA in PBS for 30 minutes. After washing with PBS, 5.0 μg/ml of monoclonal antibody to human vitronectin (Takara, Otsu, Japan) was added and incubated for 60 minutes. After washing, 2 μg/ml of goat anti-mouse IgG H&L-Alexa flour®568 (Abcam, Cambridge, UK) was also incubated for 60 minutes. Nuclei were stained with 10 μg/ml of Hoechst for 30 minutes. The expression of vitronectin was evaluated with a fluorescent microscope (BZ-9000).

### Blocking of NTHi penetration into BEAS-2B cells

Before infection with NTHi, BEAS-2B cells were pretreated with 1,000 μg/ml of heparin for 30 minutes, 10 μM of Arg-Gly-Asp (RGD) peptide for 60 minutes, or 100 μg/ml of PE^84–108^ peptide for 60 minutes. Subsequently, the cells were infected with NTHi strains for 2 hours. After treatment with gentamicin for 2 hours to kill extracellular NTHi, the amount of invaded cells and the amount of intracellular NTHi were evaluated.

### Statistical analysis

Data from multiple experiments were expressed as the mean ± standard error of the mean (SEM). Data were analyzed using a one-way ANOVA with Tukey’s post-hoc test for the comparison of three or more groups, or analyzed using a two-sided unpaired *t* test for the comparison of two groups. When one of the values was less than 5, data were analyzed using Fisher’s exact probability test. Statistical analyses were performed using SPSS Statistics version 22 (Japan IBM, Tokyo, Japan). A *p* value of < 0.05 was considered statistically significant in all tests.

## Results

### NTHi penetrates into bronchial epithelial cells

**Fig. 1:**
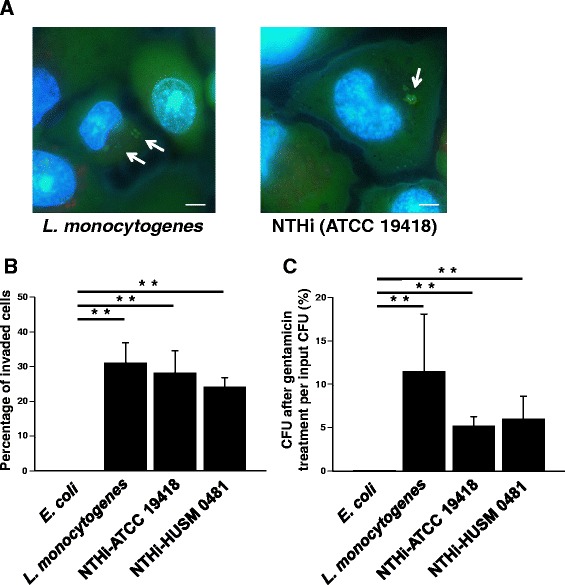
NTHi penetration of bronchial epithelial cells. BEAS-2B cells were infected with one of two NTHi strains (ATCC 19418 or HUSM 0481), *E. coli* (a negative control), or *L. monocytogenes* (a positive control) for 2 hours. After killing extracellular bacteria with gentamicin, epithelial cells and bacteria were stained with LIVE/DEAD® and Hoechst. **a** Representative fluorescence micrographs of *L. monocytogenes*, and NTHi strain ATCC 19418. White arrows show viable intracellular bacteria stained green. White bars represent 5 μm. Fluorescent micrographs were taken at 2,000× magnification. **b** The percentages of epithelial cells invaded by bacteria. **c** After killing extracellular bacteria with gentamicin, cells were lysed and the bacteria were cultured overnight. The number of colonies was counted and the percentages of CFU after gentamicin treatment per input CFU were shown. Error bars represent SEM in three independent experiments that gave similar results. *p < 0.05 and **p < 0.01

Two strains of NTHi were used in this study: a commercially available NTHi strain ATCC 19418 and a clinical isolate HUSM 0481. To confirm whether NTHi can invade bronchial epithelial cells, BEAS-2B cells were infected with NTHi for 2 hours. BEAS-2B cells were also infected for 2 hours with *E. coli* as a negative control or *L. monocytogenes* as a positive control. After killing extracellular bacteria with gentamicin, epithelial cells and bacteria were stained with LIVE/DEAD® and Hoechst and evaluated with a fluorescent microscope. Viable bacteria and cells are stained green, and dead bacteria and cells are stained red. Fluorescent micrographs showed that viable *L. monocytogenes* and NTHi strain ATCC19418 penetrate into BEAS-2B cells (representative images shown in Fig. 1a). The percentages of cells invaded by bacteria are summarized in Fig. 1b. The percentage of cells invaded by NTHi strain ATCC 19418 was 26.4 ± 4.1 % (mean ± SEM) and that by the HUSM 0481 strain was 24.0 ± 2.8 %. There were significant differences between the percentage of cells invaded by *E. coli* and that by both NTHi strains (ATCC 19418: *p* < 0.001 and HUSM 0481: *p* < 0.001 with Fisher’s exact probability test).

Next, after killing extracellular bacteria with gentamicin, cells were lysed and the bacteria were cultured overnight. Then, the number of bacterial colonies was counted. The percentage of colony number after gentamicin treatment per input bacterial number is shown in Fig. 1c. Those were 5.17 ± 1.11 % in ATCC 19418 and 5.97 ± 2.66 % in HUSM 0481. There were also significant differences between the percentage of intracellular bacteria in *E. coli* and that in both NTHi strains (ATCC 19418: *p* = 0.036 and HUSM 0481: *p* = 0.048).

### Localization of intracellular NTHi

**Fig. 2:**
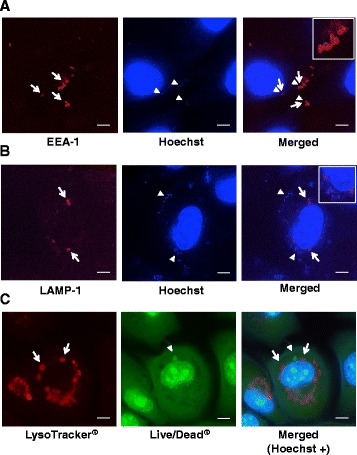
Localization of intracellular NTHi. BEAS-2B cells were infected with NTHi strain ATCC 19418 for 2 hours. After killing extracellular bacteria with gentamicin, epithelial cells and bacteria were stained with several fluorescent dyes and Hoechst (blue). **a** Representative fluorescent micrographs of intracellular NTHi (blue) and epithelial cells stained with early endosomal marker (EEA-1, red). White arrows show EEA-1-positive regions, and white arrowheads show NTHi. For a detail of merged image, see a top-right inset. **b** Representative fluorescence micrographs of intracellular NTHi (blue) and epithelial cells stained with late endosomal marker (LAMP-1, purple). White arrows show LAMP-1-positive regions, and white arrowheads show NTHi. For a detail of merged image, see a top-right inset. **c** Representative fluorescent micrographs of intracellular NTHi and epithelial cells stained with LIVE/DEAD® (green) and cells were stained with acidic lysosomal dye (LysoTracker® Red). White arrows show LysoTracker® Red-positive acidic organelles, and white arrowheads show NTHi. Fluorescent micrographs were taken at 2,000× magnification. White bars represent 5 μm. Another strain (HUSM 0481) was also tested and similar results were obtained

The localization of NTHi in epithelial cells was confirmed with a fluorescent microscope. BEAS-2B cells were infected with NTHi strain ATCC 19418 for 2 hours. After killing extracellular bacteria with gentamicin, epithelial cells and bacteria were stained with several fluorescent dyes. DNA of both intracellular bacteria and BEAS-2B cells were stained blue with Hoechst. Fluorescent micrographs at 2,000× magnification showed that intracellular NTHi (blue) localizes in early endosomes stained with EEA-1 (red) (representative images shown in Fig. 2a). However, the intracellular NTHi did not colocalize with LAMP-1 (purple; Fig. 2b), which marks late endosomes, or with acidic organelles that were marked with LysoTracker® Red (red; Fig. 2c), indicating that intracellular NTHi does not exist in late endosomes or in acidic organelles. Another strain (HUSM 0481) was also tested and similar results were obtained.

### NTHi binds to immobilized vitronectin and this Interaction is blocked by heparin

**Fig. 3:**
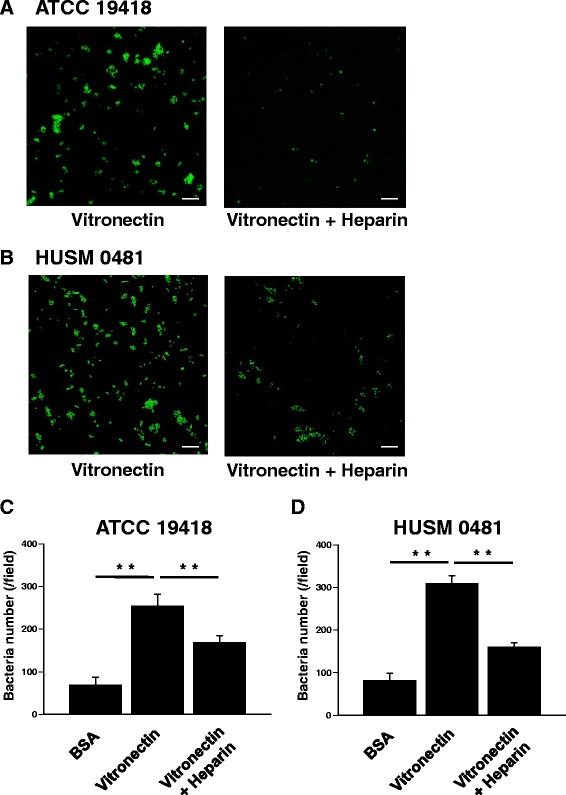
NTHi binding of immobilized vitronectin in the absence or presence of heparin. Human plasma vitronectin was bound to glass-bottomed dishes. NTHi was incubated on the plate-bound vitronectin for 30 minutes, and then the dishes were washed three times. In some experiments, plate-bound vitronectin was pretreated with heparin before incubation with NTHi. NTHi was stained with LIVE/DEAD®. Live NTHi is stained green. BSA was used as a negative control. Representative fluorescent micrographs show that both NTHi strains (ATCC 19418 (**a**) and HUSM 0481 (**b**)) attached to plate-bound vitronectin and that this attachment is blocked by heparin. White bars represent 10 μm. Summaries of the numbers of attached NTHi (per field at 1,000× magnification) are shown in (**c**) for ATCC 19418 and (**d**) for HUSM 0481. Error bars represent SEM in three independent experiments that gave similar results. **p < 0.01

Attachment to cells is important for bacterial invasion into bronchial epithelial cells. Therefore, the capacity of NTHi to bind to immobilized vitronectin was evaluated. Human plasma vitronectin was bound to glass-bottomed dishes. NTHi was incubated on the plate-bound vitronectin with or without pretreatment of 1,000 μg/ml heparin. Fluorescent micrographs showed that both NTHi strains attached to plate-bound vitronectin in the absence of heparin, but that this attachment was blocked in the presence of heparin (representative images shown in Fig. 3a and b). A summary of the numbers of attached NTHi per field at 1,000× magnification is shown in Fig. 3c (ATCC 19418) and in Fig. 3d (HUSM 0481). The number of ATCC 19418 bacteria adhered to plate-bound vitronectin was 254 ± 28/field (mean ± SEM) and this number was significantly reduced to 168 ± 16/field by blocking with heparin (Fig. 3a and c, *p* < 0.001). The number of HUSM 0481 bacteria adhered to plate-bound vitronectin was 309 ± 18/field, and this number significantly decreased to 160 ± 10/field by blocking with heparin (Fig. 3b and d, *p* < 0.001).

### BEAS-2B cells express vitronectin

We next examined whether BEAS-2B cells express vitronectin. BEAS-2B cells were stained with mouse anti-human vitronectin-antibody (primary antibody) and then with goat anti-mouse IgG antibody (secondary antibody, yellow). Nuclei were stained with Hoechst (blue). Representative fluorescent micrographs at 1,000× magnification are shown in Additional file 1: Figure S1. As a negative control, BEAS-2B cells were stained with secondary antibody alone (Additional file 1: Figure S1A). BEAS-2B cells were clearly positive for vitronectin (Additional file 1: Figure S1B). Further, there was no obvious difference in expression of vitronectin before or after NTHi infection (Additional file 1: Figure S1C).

### Intracellular invasion of NTHi is blocked by heparin, but not by RGD peptide

**Fig. 4:**
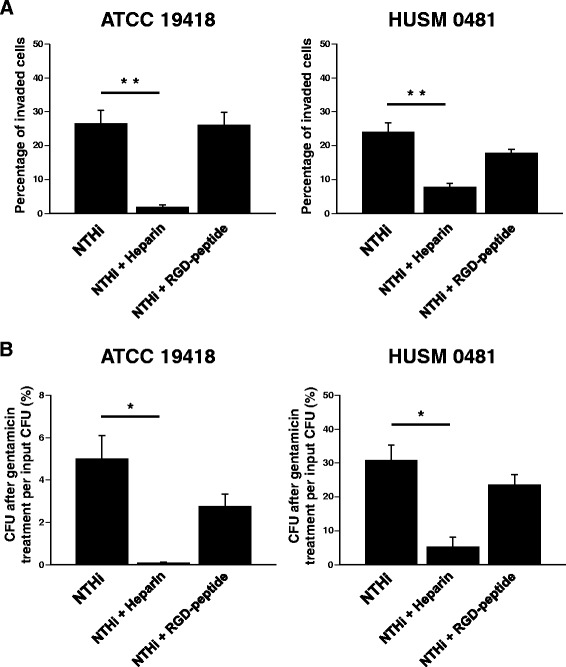
Intracellular invasion of NTHi in the presence of heparin or RGD peptide. BEAS-2B cells were infected for 2 hours with one of the two NTHi strains (ATCC 19418 or HUSM 0481), *E. coli* (a negative control), or *L. monocytogenes* (a positive control). In some experiments, cells were pretreated with heparin or RGD-peptide. **a** After killing extracellular bacteria with gentamicin, epithelial cells and bacteria were stained with LIVE/DEAD®. The percentages of BEAS-2B cells invaded by each type of bacteria are shown. **b** After killing extracellular bacteria with gentamicin and lysing the BEAS-2B cells, the bacteria were cultured overnight. The number of colonies was counted and the percentages of CFU after gentamicin treatment of cells per input CFU were shown. Error bars represent SEM in three independent experiments that gave similar results. *p < 0.05 and **p < 0.01

To determine whether intracellular invasion of NTHi is blocked by either heparin or RGD peptide, BEAS-2B cells were infected for 2 hours with one of the two NTHi strains (ATCC 19418 or HUSM 0481), *E. coli* (a negative control), or *L. monocytogenes* (a positive control) with or without pretreatment with heparin or RGD peptide. The percentage of BEAS-2B cells invaded by each type of bacteria is shown in Fig. 4a. Pretreatment with heparin, but not with RGD peptide, significantly decreased the invasion of NTHi strains (Fig. 4a, ATCC 19418: *p* < 0.001 between NTHi and NTHi + heparin, HUSM 0481: *p* < 0.012 between NTHi and NTHi + heparin). Pretreatment with heparin, but not with RGD peptide, also significantly reduced proportions of intracellular bacteria (Fig. 4b, ATCC 19418: *p* = 0.016 between NTHi and NTHi + heparin, HUSM 0481: *p* = 0.016 between NTHi and NTHi + heparin).

### Intracellular invasion of NTHi is blocked by heparin in a dose-dependent manner

Next, we evaluated how different concentrations of heparin affect the penetration of NTHi into bronchial epithelial cells. BEAS-2B cells were pretreated with several concentrations of heparin, and then these cells were infected with one of the two NTHi strains for 2 hours. The number of intracellular colonies significantly decreased as the heparin concentration increased in both strains of NTHi (Additional file 2: Figure S2A, ATCC 19418: *p* = 0.018 between 0 and 1,000 μg/mL of heparin; Additional file 2: Figure S2B, HUSM 0481: *p* = 0.01 between 0 and 1,000 μg/mL of heparin).

### Adherence of NTHi to immobilized vitronectin is blocked by protein-E peptide

**Fig. 5:**
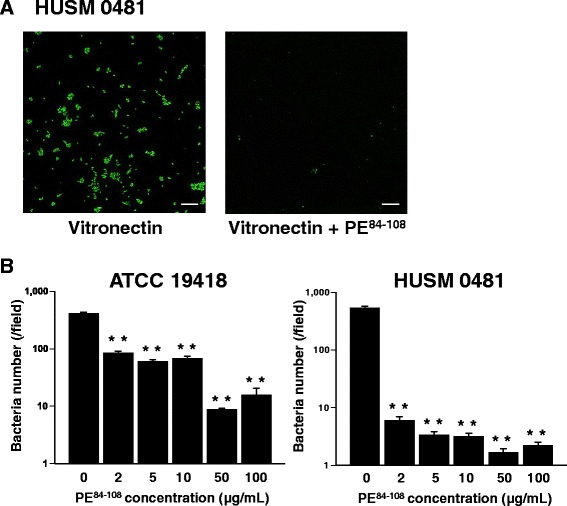
Binding of NTHi to immobilized vitronectin in the presence of protein-E peptide. Human plasma vitronectin was bound to glass-bottomed dishes. NTHi (HUSM 0481) was incubated on the plate-bound vitronectin for 30 minutes, and then the dishes were washed three times. In some experiments, plate-bound vitronectin was pretreated with protein-E peptide (PE^84–108^) before incubation with NTHi. BSA was used as a negative control. NTHi was stained with LIVE/DEAD®, and viable NTHi is stained green. **a** Representative fluorescent micrographs of NTHi incubated on plate-bound vitronectin that was untreated or pretreated with PE^84–108^. White bars represent 10 μm. **b** The number of attached NTHi per field at 1,000 × magnification that was pretreated with increasing concentrations of PE^84–108^. Error bars represent SEM in three independent experiments that gave similar results. **p < 0.01

To confirm whether bacterial protein-E is important for the ability of NTHi to adhere to vitronectin, a blocking experiment with protein-E peptide (PE^84–108^) was conducted. NTHi (HUSM 0481) was incubated on the plate-bound vitronectin with or without pretreatment with PE^84–108^. Fluorescent micrographs showed that NTHi attachment to plate-bound vitronectin was blocked by pretreatment with 100 μg/ml of PE^84–108^ (representative images shown in Fig. 5a). The number of bacteria attached to vitronectin per field at 1,000× magnification in each NTHi strain is shown in Fig. 5b. This number significantly decreased as the concentrations of PE^84–108^ increased in both strains (ATCC 19418: *p* < 0.001 between 0 and 100 μg/ml of PE^84–108^, HUSM 0481: *p* < 0.001 between 0 and 100 μg/mL of PE^84–108^).

### Intracellular invasion of NTHi is dependent on protein-E

**Fig. 6:**
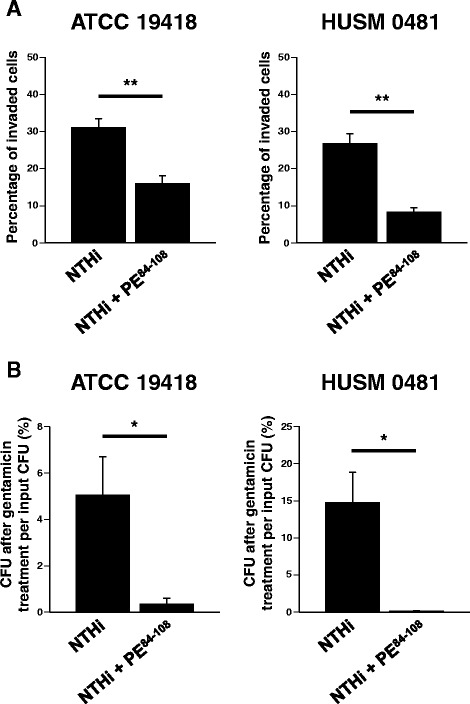
Intracellular invasion of NTHi in the absence or presence of protein-E. BEAS-2B cells were infected for 2 hours with one of the two NTHi strains (ATCC 19418 or HUSM 0481) with or without pretreatment with PE^84–108^ peptide. **a** After killing extracellular bacteria with gentamicin, epithelial cells and bacteria were stained with LIVE/DEAD®. The percentages of BEAS-2B cells invaded by each NTHi strain are shown. **b** After killing extracellular bacteria with gentamicin, cells were lysed and the bacteria were cultured overnight. The number of colonies was counted and the percentages of CFU after gentamicin treatment of cells per input CFU were shown. Error bars represent SEM in three independent experiments that gave similar results. *p < 0.05 and **p < 0.01

To determine whether protein-E is essential for NTHi penetration into bronchial epithelial cells, a blocking experiment with protein-E peptide was conducted. BEAS-2B cells were infected with one of the two NTHi strains (ATCC 19418 or HUSM 0481) with or without pretreatment with 100 μg/ml of PE^84–108^. The percentage of BEAS-2B cells invaded by each NTHi strain is shown in Fig. 6a. Pretreatment with PE^84–108^ significantly reduced the percentage of invaded cells by either strain of NTHi (Fig. 6a, ATCC 19418: *p* < 0.001, HUSM 0481: *p* < 0.01). In addition, pretreatment with PE^84–108^ significantly reduced the percentage of intracellular NTHi strains after gentamicin treatment of infected BEAS-2B cells (Fig. 6b, ATCC 19418: *p* = 0.049, HUSM 0481: *p* = 0.024).

## Discussion

**Fig. 7:**
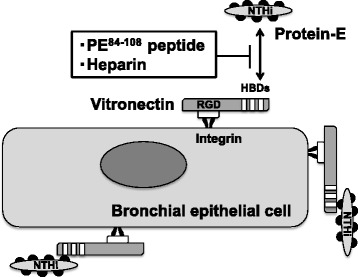
A schema of the proposed mechanism by which NTHi penetrates into bronchial epithelial cell via protein-E and vitronectin. Vitronectin has three heparin-binding domains (HBDs), which interact with NTHi. Of those HBDs, the C-terminal HBD-3 corresponds to a protein-E binding region and interacts with PE^84–108^. This interaction is blocked by heparin or PE^84–108^ peptide. Vitronectin also possesses a cell receptor binding site characterized by an Arg-Gly-Asp (RGD) sequence, which interacts with integrins on the bronchial epithelial cell surface. This protein-E-vitronectin axis seems to play a role in the adherence and penetration of NTHi into bronchial epithelial cells

Although NTHi was originally thought to be an extracellular pathogen, recent studies have indicated that NTHi breaks into bronchial epithelial cells, probably to evade the host immune system. This feature of NTHi assists the bacteria in persisting and may contribute to the intractability of COPD [6, 7]. Thus, it is important to clarify the mechanisms of NTHi intracellular invasion for the development of a novel strategy against NTHi infection. In this study, we demonstrated intracellular invasion of NTHi into bronchial epithelial cells, and we found that this invasion was able to be blocked by protein-E peptide or heparin, but not by RGD peptide. These results suggest that NTHi do not directly penetrate into bronchial epithelial cells but instead exploits protein-E and vitronectin for invasion into bronchial epithelial cells (Fig. 7). To our knowledge, this is the first report that protein-E plays a key role in the intracellular invasion of NTHi as well as in NTHi attachment to bronchial epithelial cells.

Although there have been several studies reporting possible mechanisms of NTHi adhesion to epithelial cells, the mechanisms of the intracellular invasion remained poorly understood. NTHi has several adhesion molecules; *Haemophilus* adhesion and penetration protein (Hap) [18, 19], high-molecular-weight proteins 1 and 2 (HMW1 and HMW2) [20], and *Haemophilus influenzae* adhesin (Hia) [21], protein-E [22], and protein-F [23] have all been shown to mediate bacterial adherence to bronchial epithelial cells. In terms of penetration of NTHi into bronchial epithelial cells, a process caused by cytoskeletal rearrangement accompanied with actin and microtubule polymerization allows NTHi to invade cells. Several mechanisms of direct invasion of *H. influenzae* into bronchial epithelial cells have been reported, including (1) macropinocytosis [24], (2) platelet-activating factor (PAF) receptor via NTHi phosphorylcholine on lipooligosaccharide [25, 26], (3) β-glucan receptor [27], and (4) α5β1-integrin [11]. These mechanisms of NTHi penetration are attributed to direct interactions between NTHi and epithelial cells. However, mechanisms for indirect invasion of bacteria have recently been reported; *Haemophilus* surface fibril (Hsf) of *H. influenzae* type b (Hib) was shown to be involved in the intracellular invasion of Hib via binding to vitronectin [28]. Hsf is a major trimeric autotransporter adhesin exclusively expressed in encapsulated *H. influenzae* strains such as Hib. Hsf binds to the C-terminal amino acids 352–374 in the heparin-binding domains (HBDs) of vitronectin. Vitronectin bound to Hsf increases the adherence and internalization of Hib into bronchial epithelial cells [28]. Because we used NTHi, but not Hib, it is unlikely that Hsf is involved in the intracellular invasion observed in this study. Hia, which has homology with Hsf in Hib, is a trimeric autotransporter found in NTHi. However, Hia is present in only approximately 25 % of clinical NTHi isolates [29], and so far, there has been no report to show that Hia is involved in intracellular invasion of NTHi. Here, we report a novel mechanism of NTHi intracellular invasion that involves an interaction between NTHi protein-E and vitronectin. We believe that protein-E, but not Hia, plays a pivotal role in this NTHi invasion mechanism.

Protein-E is a low molecular-mass (16 kDa) outer membrane lipoprotein and is highly conserved in both NTHi and encapsulated *H. influenzae* strains [17, 22]. Protein-E has been reported to bind serum vitronectin and to reduce membrane attack complex (MAC)-induced lysis of NTHi [14, 16, 17]. Protein-E has also been shown to bind immobilized vitronectin [14]. Vitronectin is an important component of extracellular matrix and is related to bacterial serum resistance and adhesion [15]. Binding of vitronectin to vitronectin-binding proteins on bacterial surface is able to block C5b-7 complex formation and C9-polymerization, which constitutes MAC, and protects the bacteria from MAC-induced lysis [14, 15]. Therefore, the binding between vitronectin and bacteria through vitronectin-binding proteins of bacteria, such as protein-E, is essential for this process.

Vitronectin has three HBDs, which interact with various bacteria. *H. influenzae* binds to vitronectin through the HBDs, and their binding is blocked by heparin [13]. Among the HBDs, the C-terminal HBD-3 of vitronectin corresponds to a protein-E binding region (amino acids 353–363) [16]. The binding domain of protein-E to vitronectin includes amino acids 84–108 (PE^84–108^), and this peptide has been reported to block binding between NTHi and vitronectin [14]. In agreement with these results, the present study showed that the PE^84–108^ peptide could block adhesion of NTHi to plate-bound vitronectin and that pretreatment with this peptide prevented NTHi invasion into epithelial cells. Moreover, we demonstrated that BEAS-2B cells abundantly express vitronectin, and that heparin and PE^84–108^ peptide pretreatment significantly reduced NTHi intracellular invasion. These results show that the interaction between NTHi protein-E and vitronectin plays an important role in NTHi intracellular invasion (Fig. 7). In this study, heparin and PE^84–108^ peptide significantly, but not completely, diminished the NTHi intracellular invasion. Thus, other mechanisms may also be involved in this process. For example, NTHi protein-F has also been reported to bind vitronectin [23]. Protein-F promotes vitronectin-dependent bacterial adhesion to the cell surface, although the binding strength of protein-F to vitronectin is much weaker than that of protein-E.

Vitronectin has a cell receptor binding site characterized by an RGD sequence that interacts with cell surface integrins [15]. Therefore, an RGD peptide should inhibit the binding of vitronectin to integrins on bronchial epithelial cells. *Streptococcus pneumoniae* has been reported to exploit vitronectin and αvβ3 integrin for its adherence and intracellular invasion to A549 lung alveolar epithelial cells [30]. However, in our study, RGD peptide did not block the intracellular invasion of NTHi. Our fluorescent study on BEAS-2B cells revealed an intense expression of vitronectin on the cell surface as well as in the cytoplasm. Vitronectin may already be bound to integrins on the epithelial cell surface, which would prevent the intracellular invasion from being affected by the RGD peptide.

In this study, intracellular NTHi localized in early endosomes stained with EEA-1, but not in late endosomes stained with LAMP-1 or in acidic organelles. These results were different from those in previous study, which showed NTHi mainly located in LAMP-1-positive compartment [4]. This discrepancy may be due to the difference in the types of epithelial cells used and in the time points after infection.

## Conclusions

The present study demonstrated that the intracellular invasion of NTHi into bronchial epithelial cells is mediated by the interplay between protein-E on NTHi and vitronectin on bronchial epithelial cells. Our findings provide novel information about the NTHi-epithelial cell interaction leading to NTHi entry into these cells. The protein-E-vitronectin axis may become a novel therapeutic target for NTHi infection. Further study is needed to achieve this goal in clinical practice.

## Additional files

Additional file 1: Figure S1. Expression of vitronectin in BEAS-2B cells. BEAS-2B cells were stained with mouse anti-human vitronectin-antibody (primary antibody) and then with goat anti-mouse IgG antibody (secondary antibody, yellow). Nuclei were stained with Hoechst (blue). Representative fluorescent micrographs at 1,000× magnification are shown. (A) BEAS-2B cells were stained with the secondary antibody without the primary antibody. (B) Uninfected BEAS-2B cells. (C) BEAS-2B cells infected with NTHi. White bars represent 10 μm. (PPTX 4302 kb) Additional file 2: Figure S2. Intracellular invasion of NTHi in the presence of increasing dose of heparin. BEAS-2B cells were pretreated with several concentrations of heparin. These cells were infected for 2 hours with one of the two NTHi strains ((A) ATCC 19418 or (B) HUSM 0481). After killing extracellular bacteria with gentamicin, the BEAS-2B cells were lysed. The number of colonies was counted and the percentages of CFU after gentamicin treatment of cells per input CFU were shown. Error bars represent SEM in three independent experiments that gave similar results. **p* < 0.05. (PPTX 72 kb)

## Abbreviations

NTHi: nontypeable *Haemophilus influenza*
RGD: Arg-Gly-Asp
HBDs: heparin-binding domains
HMW: high-molecular-weight proteins
DNA: deoxyribonucleic acid
COPD: chronic obstructive pulmonary disease
SEM: standard error of the mean
