# Supplementary figures and images for: Nontypeable Haemophilus influenzae exploits the interaction between protein-E and vitronectin for the adherence and invasion to bronchial epithelial cells
Source: BMC Microbiol. 2015 Nov 14;15:263. doi: 10.1186/s12866-015-0600-8 (PMC4647820; doi:10.1186/s12866-015-0600-8)

## Slide 1
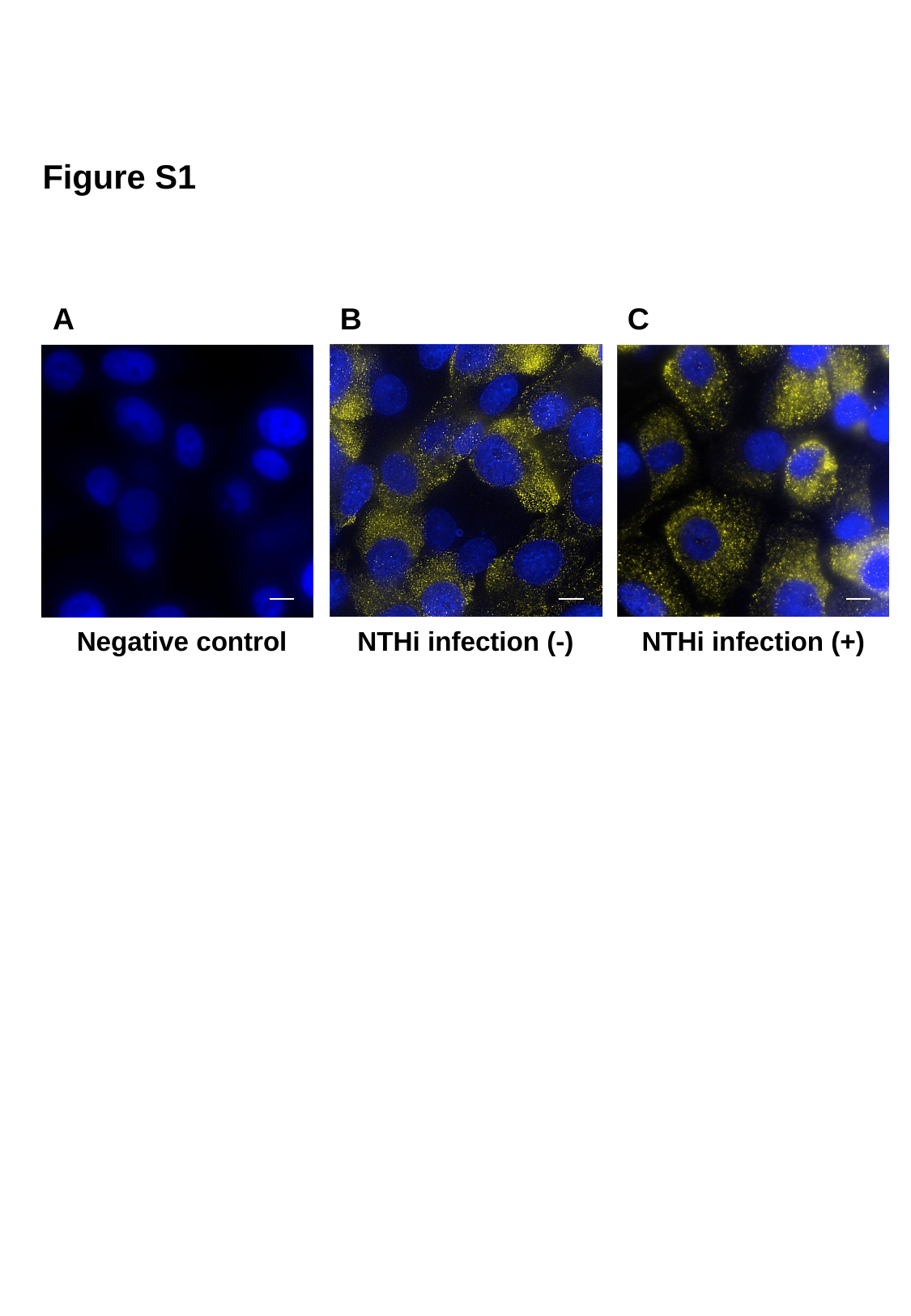

Figure S1
A
B
C
Negative control
NTHi infection (-)
NTHi infection (+)

Supplement: Additional file 1: Figure S1. — Expression of vitronectin in BEAS-2B cells. BEAS-2B cells were stained with mouse anti-human vitronectin-antibody (primary antibody) and then with goat anti-mouse IgG antibody (secondary antibody, yellow). Nuclei were stained with Hoechst (blue). Representative fluorescent micrographs at 1,000× magnification are shown. (A) BEAS-2B cells were stained with the secondary antibody without the primary antibody. (B) Uninfected BEAS-2B cells. (C) BEAS-2B cells infected with NTHi. White bars represent 10 μm. (PPTX 4302 kb) [file 12866_2015_600_MOESM1_ESM.pptx]
