# Supplementary material for: Nontypeable Haemophilus influenzae exploits the interaction between protein-E and vitronectin for the adherence and invasion to bronchial epithelial cells
Source: BMC Microbiol. 2015 Nov 14;15:263. doi: 10.1186/s12866-015-0600-8 (PMC4647820; doi:10.1186/s12866-015-0600-8)
Supplement: Additional file 2: Figure S2. — Intracellular invasion of NTHi in the presence of increasing dose of heparin. BEAS-2B cells were pretreated with several concentrations of heparin. These cells were infected for 2 hours with one of the two NTHi strains ((A) ATCC 19418 or (B) HUSM 0481). After killing extracellular bacteria with gentamicin, the BEAS-2B cells were lysed. The number of colonies was counted and the percentages of CFU after gentamicin treatment of cells per input CFU were shown. Error bars represent SEM in three independent experiments that gave similar results. *p < 0.05. (PPTX 72 kb) [file 12866_2015_600_MOESM2_ESM.pptx]

## Slide 1
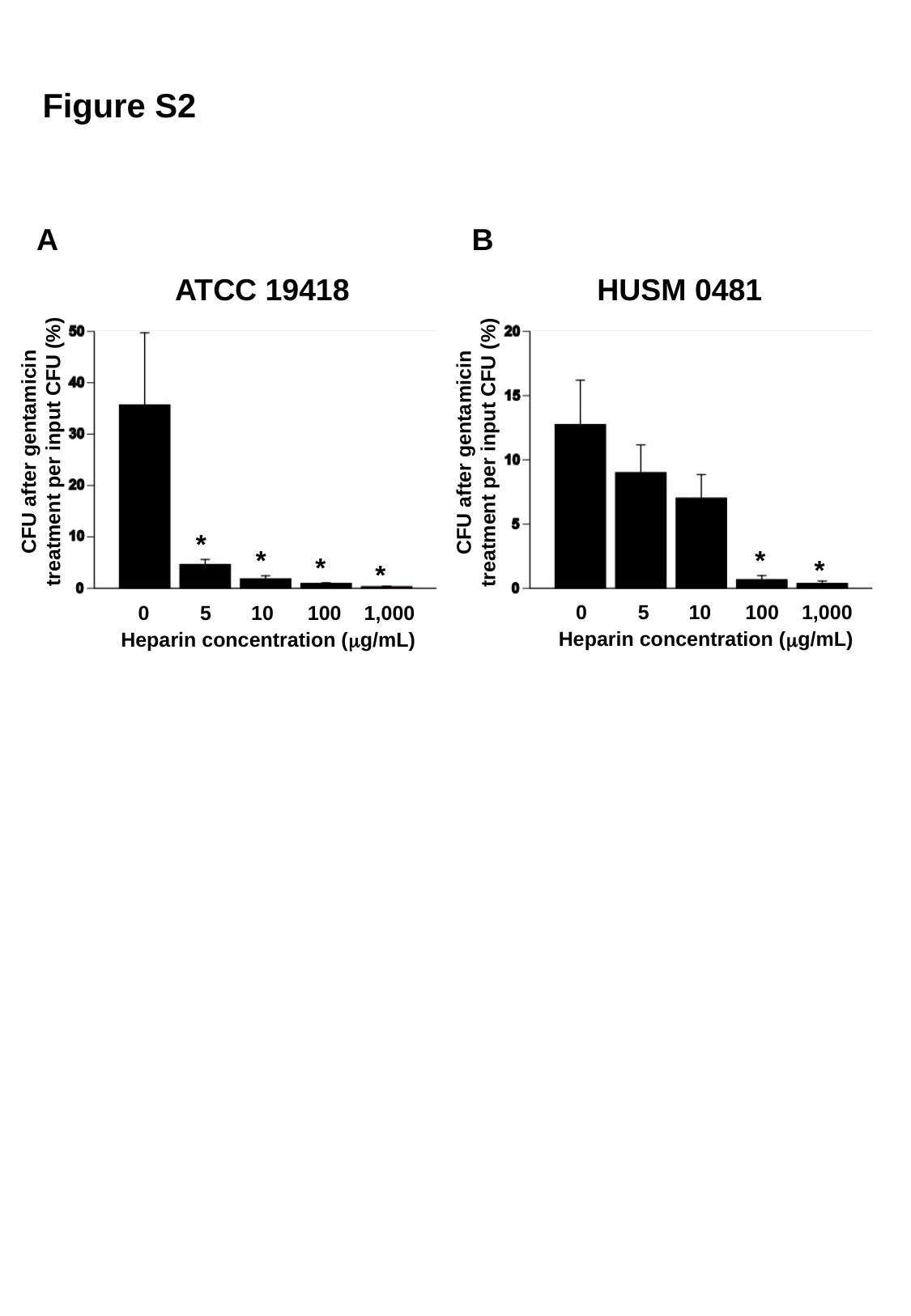

Figure S2
A
B
ATCC 19418
HUSM 0481
CFU after gentamicin treatment per input CFU (%)
CFU after gentamicin treatment per input CFU (%)
*
*
*
*
*
*
 0 5 10 100 1,000
 0 5 10 100 1,000
Heparin concentration (μg/mL)
Heparin concentration (μg/mL)
